# Microsurgery Resection of Intrinsic Insular Tumors via Transsylvian Surgical Approach in 12 Cases

**DOI:** 10.3969/j.issn.2095-3941.2012.01.008

**Published:** 2012-03

**Authors:** Peng Wang, Ming-can Wu, Shi-jie Chen, Xian-ping Xu, Yong Yang, Jie Cai

**Affiliations:** 1 Department of Neurosurgery, Neuroscience Center, Nanjing Medical University, Affiliated Wuxi No.2 Hospital, Wuxi 214002, China; 2 Department of Neurosurgery;; 3 Department of Radiology, The First People’s Hospital of Jingzhou, Jingzhou 434000, China

**Keywords:** microsurgery, cerebral cortex, glioma, diffusion tensor imaging

## Abstract

**Objective:**

To investigate the clinical characteristics, operative methods, and diffusion tensor imaging (DTI) in the resection of intrinsic insular gliomas via transsylvian approach.

**Methods:**

From June 2008 to June 2010, 12 patients with intrinsic insular gliomas were treated via transsylvian microsurgical approach, with preoperative magnetic resonance imaging diffusion tensor imaging (MR DTI) evaluation. The data of these patients were retrospectively analyzed.

**Results:**

All patients had astrocytoma, including 8 patients of Grades I to II, 2 patients of Grades III to IV, and 2 patients of mixed glial tumors. The insular tumors were completely removed in 9 patients, whereas they were only partially removed from 3 patients. No death was related to the operations. Two patients had transient aphasia, 2 experienced worsened hemiplegia on opposite sides of their bodies, and 2 had mild hemiplegia and language function disturbance.

**Conclusions:**

Most of the insular gliomas are of low grade. By evaluating the damage of the corticospinal tract through DTI and using ultrasonography to locate the tumors during operation, microsurgery treatment removes the lesions as much as possible, protects the surrounding areas, reduces the mobility rate, and improves the postoperative quality of life.

## Introduction

The insular lobe is a region in the center of the cerebral hemisphere found deep in the Sylvian fissure, covered by the opercula of the frontal, parietal, and temporal lobes. The insular lobe is responsible for gustatory, olfactory, auditory, visual, and somatosensory functions. Surrounding it are vital branches of cerebral middle artery (CMA) and nerves, such as Broca’s area, basal ganglion, internal capsule, and other powerful brain regions. Performing an en bloc resection through these vital vessels and nerves is a challenge for neurosurgeons and poses a significant risk to patients. The microsurgery of the insular gliomas via transsylvian approach has improved with the development of the microneurosurgery technique and the knowledge of microanatomy function of the insular area. The success of resection of the insular lesion depends on the microneurosurgeon’s skills, the microanatomic knowledge of this area, medical technology, and equipment used ^[^^1^^–^^3^^]^. From 2008 to 2010, 12 cases have been treated via transsylvian microneurosurgery in our hospital, where a combination of microsurgery technique and the new fiber-tracking tensor imaging (MR DTI) for pre- and post-operation assessments was used.

## Patients and Methods

### Patients

There were 12 cases of insular gliomas from 2008 to 2010 in our department. The patients comprised 5 males and 7 females, ranged from 36 years old to 58 years old, with an average age of 40.5. Four cases complained of headache and dizziness, 8 experienced seizures, 2 experienced paralysis, and 2 had loss of memory. All cases were examined through MRI and MR DTI. Seven cases had lesions in the left hemisphere, whereas 5 cases in the right hemisphere.

According to the mechanism and development of the limbic system and insular gliomas in different opercula, the researchers ^[^^4^^, ^^5^^]^ proposed four types of cases connected with the insular lobe. The 12 cases observed in our department were as follows: 3 cases were type I with the whole lesion only restricted in the insular lobe or a part of it; 2 cases were type II with the lesion in both insular and frontal opercula lobe; 4 cases were type III with the lesion is located at the medial temporal base and the insular lobe; and 3 cases were type IV with the lesion in the insular, fronto-orbital cortex, or temporopolar area. The tumor diameter ranged from 2.5 cm to 6.0 cm.

### MRI and MR DTI examination

**Figure 1-15:**
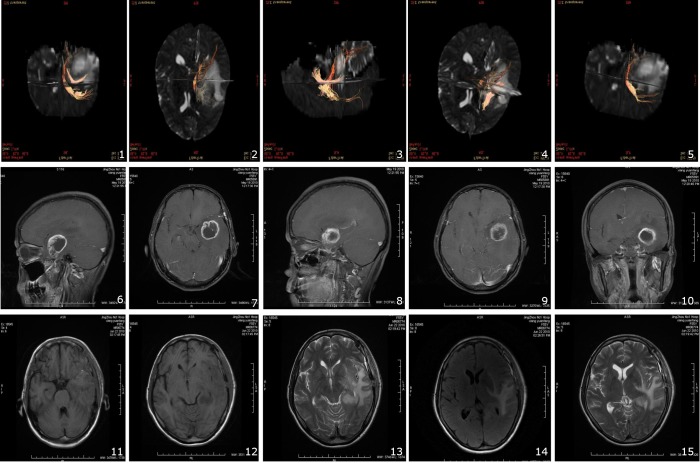
MR DTI (Figure 1-5) can assess the completeness and consistency of the white matter fibers, “depression, edema, infliction, and damage” by the tumor. The pyramidal tracts and the deep white matter were partially invaded and damaged. MRI (Figure 6-15), preoperative axial (Figure 7, 9), sagittal (Figure 6, 8), and coronal (Figure 10) T1-weighted enhancecd image showed tumor were hyper-signal with sharp border. Marked edemas surrounded the location at the medial temporal base and the insular lobe. Postoperative axial T1-weighted image (Figure 11, 12), T2-weighted image (Figure 13, 15) and T2 flair image (Figure 14) showed total tumor removal with mild edema surrounding.

Most of the insular tumors were hyposignal in T1-weighted images but were hyper-signal in both T2-weighted image and contrast image with sharp border. The pre-operative MR DTI clearly showed the anatomical relation between the pyramidal tracts and the lesions, how much the white matter fibers were pushed away by the lesion, and the damage in the pyramidal tracts (**Figure 1**** and ****2**). The available white matter fibers could also be located in the computer to predict further prognosis and to decide suitable surgical methods for the best resection of the tumors.

### Microsurgery treatment

In all cases, surgery was done in a lateral position. Pterional transsylvian craniotomy was performed, with the fronto-temporal skin and temporalis muscle flap raised. The sylvian fissure was first opened under a microscope at the anterior 2/3 length. Cotton was used to protect superficial sylvian veins. Vital vessels and the surroundings were sometimes difficult and complicated to identify because of the distorted anatomical structures induced by the tumor. The sylvian fissure was opened wide, and the temporal branches of the superficial sylvian veins were sacrificed to protect the branches in the frontal area. This method could minimize postoperative venous thrombosis in the frontal lobe, particularly the frontal operculum and Broca’s area. The middle cerebral artery (MCA) and its branches were identified. In case there were no clear vascular structures, the origin of MCA from the internal carotid artery was found, taking care not to damage the medial side of its first segment. The surgery was done between the frontal and temporal main branches of the MCA. By debulking the center of the tumor or cutting several pieces off, the tumor was safely resected without damaging the internal capsule, the putamen, or the globus pallidus.

## Results

DTI showed that 2 cases had little damage on the pyramidal tracts, 3 cases had pyramidal tracts severely pushed away by the lesion, and 7 cases had damaged pyramidal tracts that partially invaded the deep white matter. The insular gliomas were removed from 9 patients and partially removed in 3 patients. Partial resections were done either because the tumor was deep inside the medial area, already infiltrated the internal capsule and thalamus or was close to the lenticulostriate arteries. No death occured. However, 2 patients experienced transient aphasia, 2 had worsen hemiplegia on opposite sides of the body, and 2 experienced mild hemiplegia and disturbance of language function. After the operation, all patients took pills to prevent epilepsy. Pathological outcomes showed that all patients had astrocytoma, including 8 patients of Grades I to II, 2 patients of Grades III to IV, and 2 patients who had mixed glial tumors. Patients with Grades III and IV tumors received adjuvant treatment with radiotherapy and chemotherapy.

## Discussion

The insular lobe is a complex structure constituting the anatomic, cytoarchitectonic, and functional interfaces between the allocortex and the neocortex. This area is part of a larger system that includes the fronto-orbital, temporopolar, and insular regions. The area constitutes the paralimbic system or mesocortex. The insular lobe is involved in the gustatory, olfactory, auditory, and vestibular senses, motor integration, and motor planning of speech ^[^^2^^, ^^3^^]^.

The whole blood supply of the insular lobe comes from MCA, which has three branches: cortex arteries from the M1, lateral branches of the temporal lobe, and medial branches of the frontal lobe. Lenticulostriate arteries (LLAs) come from the inferior middle part of M1 and are lateral to the anterior perforators. They are the suppliers of the putamen, globus pallidus, head and body of caudate nucleus, internal capsule, and the lateral part of the anterior commissure. Damage of the main branches could lead to infarction. There is an 11 mm area between the LLAs and the liminen insular; without perforating the arteries, this area can be a possible site for resection ^[^^6^^, ^^7^^]^.

The reported insular tumors are mostly low-grade gliomas or mesogliomas, and they may slowly grow outward ^[^^1^^]^. Seizures are considered one of the most common clinical symptoms. Although the mechanism of insular epilepsy is still uncertain, results are unsatisfactory in only 42.6% of cases after temporal lobectomy and insulectomy, compared with 83.3% of patients without insular ablation. Based on this data, insular epilepsy may have multiple anatomical connections with the olfactory cortex, amygdala, entorhinal cortex, cingulate gyrus, and hippocampus. In addition, ictal insular symptoms, such as visceral, gustatory, and somatosensory signs, are attributed to the mesial temporal lobe. Seizures do not only originate from the insular cortex but also from other vital areas ^[^^7^^,^^8^^]^. In our study, 8 cases (66.7%) experienced seizures after operation, 2 had partial seizures, and 6 did not experience seizure attacks.

The MR DTI examination can help diagnose insular gliomas using the volume, location, and anatomical connection with the surrounding areas. The MRI T2 shows the bond between the deep basal ganglion and the internal capsule. The neurosurgeon needs to identify the clear margin and the depth of resection, and ascertain whether the tumor infiltrates the internal capsule or other important structures ^[^^9^^]^. MR DTI can assess the completeness and consistency of the white matter fibers, “depression, edema, infliction, and damage” of the tumors (**Figure 1**** to ****5**), and can distinguish the corticospinal tract, optic radiations, motor function areas, and language tracking areas ^[^^10^^, ^^11^^]^. Tractography is helpful for the design of surgical approaches, the identification of areas to be resected, and the prediction of clinical function (**Figure 6**** to ****15**).

Studies found no significant difference in the survival time between patients treated with microsurgery and those treated with conservative methods ^[^^4^^]^. This result is attributed to the deep sylvian fissure inside the insular gliomas, the vital surrounding areas, and severe postoperative complications, such as aphasia and hemiplegia, especially for patients of malignant insular gliomas. However, as microneurosurgery techniques are developed and more knowledge of the microanatomic function of the insular area are obtained, and because insular gliomas grow mostly in the gluconeogenesis cortex, surgeons nowadays prefer total resection of insular gliomas without damaging normal surrounding nerves. Patients treated with this method had longer survival and better quality of life ^[^^12^^-^^14^^]^. The first series of large surgeries of insular tumors via transsylvian approach were proposed by Yasargil et al. ^[^^5^^]^. This transsylvian approach is now widely accepted and performed all over the world. The rate of total resection of insular gliomas and patients’ postoperative quality of life have improved significantly. In this study, 12 resections were installed in a lateral position with the head turned 30°. Pterional transsylvian craniotomy was performed under a microscope. Before splitting the sylvian fissure, cerebrospinal fluid was released to reduce intracranial pressure. Ultrasonography was used to locate the tumors and the branches of the MCA, and subpial resection was performed to avoid the MCA and its branches.

Pterional transsylvian approach can open the sylvian fissure wide to expose the insular glioma with reduced intracranial pressure, without damaging the temporal and frontal lobes. Ultrasonography can identify the depth of the tumor for microsurgery resection. The small arteries around the tumor should be protected because they can still perforate arteries that supplies the internal capsule and thalamus. Protecting these arteries can reduce postoperative complications.
